# The Mental Hospitals Association

**Published:** 1921-09-17

**Authors:** 


### The Mental Hospitals Association.

The West Riding of Yorkshire Asylums Board has decided to withdraw from membership of the Mental Hospitals Association, of which the third annual meeting was recently held. Mr. J. W. Turner, who was the delegate to that meeting, reported to the Asylums Board that the proceedings had been interesting and satisfactory, and it had been proposed that the law should be altered to allow incipient cases to be treated without certification in asylums. A clause to this effect had been inserted in the Administrative Provisions Bill, but the Bill dealt with so many matters that it had been dropped. Mr. B. Crowther proposed that the Board withdraw its membership. After three years he himself was still puzzled about the Association's constitution, rules, and probable expenditure. To support the Association, in his opinion, was waste of time and money. It had met seven or eight times during the year to consider the remuneration of the staffs, but not once to consider the welfare of the patients. The proposed alteration of the law and the "biased work of the Conciliation Committee" were its sole accomplishments. Mr. H. Lockwood defended the Association. It was likely to be useful in co-ordinating and uniting asylum authorities. Because there was someone representative of trade unionism at the head of the Association, and Mr. Crowther was strongly opposed to collective bargaining, he called the organisation biased. Notwithstanding other speeches in support of the Association, Mr. Crowther's motion to withdraw from membership was carried by 11 votes to 6. At the same meeting a special committee was appointed to consider and report on wages and conditions of employment.

